# Comparison of Regression Methods for Modeling Intensive Care Length of Stay

**DOI:** 10.1371/journal.pone.0109684

**Published:** 2014-10-31

**Authors:** Ilona W. M. Verburg, Nicolette F. de Keizer, Evert de Jonge, Niels Peek

**Affiliations:** 1 Department of Medical Informatics, Academic Medical Center, University of Amsterdam, Amsterdam, the Netherlands; 2 Department of Intensive Care, Leiden University Medical Center, Leiden, the Netherlands; 3 Health eResearch Centre, Centre for Health Informatics, University of Manchester, Manchester, United Kingdom; D'or Institute of Research and Education, Brazil

## Abstract

Intensive care units (ICUs) are increasingly interested in assessing and improving their performance. ICU Length of Stay (LoS) could be seen as an indicator for efficiency of care. However, little consensus exists on which prognostic method should be used to adjust ICU LoS for case-mix factors. This study compared the performance of different regression models when predicting ICU LoS. We included data from 32,667 unplanned ICU admissions to ICUs participating in the Dutch National Intensive Care Evaluation (NICE) in the year 2011. We predicted ICU LoS using eight regression models: ordinary least squares regression on untransformed ICU LoS,LoS truncated at 30 days and log-transformed LoS; a generalized linear model with a Gaussian distribution and a logarithmic link function; Poisson regression; negative binomial regression; Gamma regression with a logarithmic link function; and the original and recalibrated APACHE IV model, for all patients together and for survivors and non-survivors separately. We assessed the predictive performance of the models using bootstrapping and the squared Pearson correlation coefficient (R^2^), root mean squared prediction error (RMSPE), mean absolute prediction error (MAPE) and bias. The distribution of ICU LoS was skewed to the right with a median of 1.7 days (interquartile range 0.8 to 4.0) and a mean of 4.2 days (standard deviation 7.9). The predictive performance of the models was between 0.09 and 0.20 for R^2^, between 7.28 and 8.74 days for RMSPE, between 3.00 and 4.42 days for MAPE and between −2.99 and 1.64 days for bias. The predictive performance was slightly better for survivors than for non-survivors. We were disappointed in the predictive performance of the regression models and conclude that it is difficult to predict LoS of unplanned ICU admissions using patient characteristics at admission time only.

## Introduction

Hospitals face continuous pressure to improve quality and reduce costs. The care provided by intensive care units (ICUs) is complex and the associated costs are high, so ICUs are particularly interested in assessing, comparing and improving their performance. To do this they often use case-mix adjusted outcome measures, such as in-hospital mortality and length of stay (LoS) on the ICU. ICU LoS can serve as an indicator for efficiency of care as it is strongly related to ICU costs. Prognostic models, such as APACHE II [1], [2], SAPS II [3] and APACHE IV [2], [4] have been proposed and widely implemented to adjust hospital mortality for ICU case-mix. However, the predictive performance for LoS of existing models is poor [5]–[8] and little consensus exists on the best method for predicting this outcome.

Existing models for predicting ICU LoS, such as the commonly used APACHE IV [6] model, make use of ordinary least squares (OLS) regression on untransformed ICU LoS [9], [10] or log-transformed ICU LoS [11]–[13]. These models make no distinction between ICU survivors and non-survivors, although the association between patient characteristics and ICU LoS is often strikingly different for these two groups. For instance, comorbidities tend to prolong the LoS of survivors, while accelerating death in non-survivors. The fact that ICU LoS is often positively skewed also causes problems for OLS regression, which assume symmetrical error distributions. Although, regression methods for modeling positively skewed data have been proposed [14], these models have not been used to predict ICU LoS. Previously, researchers have examined the performance of a range of regression models to analyze hospital LoS in a cohort of patients undergoing coronary artery bypass graft (CABG) surgery [15]. Patient who experienced an unplanned admission to the ICU, are more heterogeneous in terms of case-mix than those admitted following CABG surgery. Patients undergoing elective surgery (planned admissions) require a different ICU indication, often monitoring for a fixed ICU LoS, than patients, who experience an unplanned ICU admission. Furthermore, ICU mortality is higher for patients with an unplanned ICU admission than for CABG patients.

In this study, we investigated the feasibility of predicting individual patient LoS following an unplanned admission to the ICU for medical reasons or following emergency surgery. These patients form an heterogeneous population with a substantial mortality rate. We developed the prognostic models for all patients together and for survivors and non-survivors separately. Patients, who leave the ICU alive are often discharged at set times of day, leading to a multimodal distribution of observed ICU LoS. Hence, we investigated whether cyclical terms (cosine and sine functions of discharge time) [16] increased the predictive power of our models. We compared OLS regression, generalized linear models (GLMs), and Cox proportional hazards (CPH) regression on data from the NICE ICU registry [17]. We included patient characteristics, but no structural or organizational characteristics of the ICUs, so that our models could potentially be used to correct for case-mix when comparing institutions.

## Materials and Methods

### Data

Since 1996, the Dutch National Intensive Care Evaluation (NICE) registry has collected data on intensive care patients in the Netherlands [17]. The registry collects data on the severity of illness from the first 24 hours of a patient's ICU admission, including the diagnosis, Glasgow Coma Scale (GCS), physiological and laboratory values needed to calculate severity of illness score such as the APACHE II [1], [2], SAPS II [3] and APACHE IV [2], [4] scores. In addition, NICE registers ICU and hospital LoS and mortality. To ensure that the data are of a high quality, the data are subjected to quality checks, onsite data quality audits take place and data collectors participate in training sessions.

We obtained permission from the secretary of the NICE board, Dr. D.W. de Lange, email: info@stichting-nice.nl, to use data from the NICE registry at the time of the study. The NICE board assesses each application to use the data on the feasibility of the analysis and whether or not the confidentiality of patients and ICUs will be protected. To protect confidentiality, raw data from ICUs is never provided to third parties. For the analyses described in this paper, we used an anonymized dataset. The use of anonymized data does not require informed consent in the Netherlands. The data are officially registered in accordance with the Dutch Personal Data Protection Act.

The data in this study was obtained from all medical and unplanned surgical admissions between January 1^st^ 2011 and December 31^st^ 2011 to 83 ICUs, representing more than 90% of all ICUs in the Netherlands. Of the ICUs, 52 (63%) were general, 25 (30%) teaching and 6 (7%) university-affiliated hospitals. We applied the APACHE IV exclusion criteria [2] and excluded patients younger than 16 on admission to the ICU; with ICU LoS shorter than four hours; with hospital LoS longer than 365 days; with unknown hospital discharge date; who died before ICU admission; readmissions; coming from another ICU; with unknown ICU admission type; or with unknown diagnosis, burns or following a transplant. In addition, we excluded patients, who were discharged to another ICU, as their observed ICU LoS was truncated, and patients with missing values for model covariates.

### Definition of length of stay

We defined ICU LoS as the period between ICU admission date and time and ICU discharge date and time. We rounded ICU LoS to the nearest number of whole hours to enable us to perform Poisson and negative binomial regression. We present the results of the validation of our models in days, with decimals representing fractional days.

### Regression methods

We used eight regression methods to predict ICU LoS: 1) OLS regression on untransformed ICU LoS, 2) OLS regression on ICU LoS truncated at 30 days; 3) OLS regression on log-transformed ICU LoS; 4) a GLM with a Gaussian distribution and a logarithmic link function; 5) Poisson regression; 6) negative binomial regression; and 7) Gamma regression with a logarithmic link function; 8) CPH regression. In addition, we predicted LoS using the APACHE IV model in its original form and recalibrated on our data [18]. When predicting ICU LoS using an OLS regression model, we replaced negative values with zeros, since ICU LoS is always positive. We present the details of the statistical background of these methods in text S1.

### Survival status

We developed the prognostic models once using data from all patients and once using data from ICU survivors and ICU non-survivors separately. We defined survivors and non-survivors by their survival status at discharge from the ICU.

### Variable selection

We initially included a set of patient characteristics, presented in Table S1, previously shown to be associated with ICU LoS [1], [2], [5], [19] in each of the models. The models were subsequently simplified using stepwise backward selection with the Akaike Information Criterion. We compared univariate regression models, in which we included age and APACHE IV physiology score (APS) as continuous covariates and as natural regression splines [20] with two to ten degrees of freedom. As a result of these analyses, we included age and APS in further models using natural regression splines with three degrees of freedom.

### Cyclical terms

For survivors of ICU treatment, patient discharge often takes place at set times during the day, which leads to a multimodal distribution of observed ICU LoS, with a period of one day. However, predictions could be biased when predicting ICU LoS using regression methods, which typically assume a unimodal distribution. Hence, we also developed models with cyclical terms for discharge time as covariates [16]. These cyclical terms are presented in more detail in text S2.

Overall, we applied eight (three OLS models, four GLMs, one CPH model) different regression methods, with and without cyclical terms to the entire dataset and to separate datasets for survivors and non-survivors. Hence, in total we developed 32 regression models and calculated predictive performance for all patients using these 32 models and the original and recalibrated APACHE IV models.

### Performance assessment

To evaluate each model's ability to predict ICU LoS, we examined four measures of predictive performance based on differences between predicted and observed ICU LoS. These were: 1) squared Pearson correlation coefficient (R^2^) [21]; 2) root mean squared prediction error (RMSPE); 3) mean absolute prediction error (MAPE); 4) prediction bias [9], [15]. We describe these measures in more depth in text S3.

The R^2^ is the fraction of variance in observed ICU LoS that is explained by a model. It ranges from 0 to 1, where higher values correspond to better predictions. The RMSPE represents the mean residual, or unexplained, standard error of predictions obtained using a model. Because of the extreme skewedness of the distribution of ICU LoS, the RMSPE increases quickly if a long LoS are erroneously predicted to be short or vice versa. In other words, a single mistake by the model may dominate the RMSPE. Therefore, we also present the MAPE, which does not have this limitation. Finally we assess whether a model's predictions systematically deviate from observed LoS values, using the prediction bias. For RMSPE, MAPE and prediction bias, lower values correspond to better.

We assessed the performance of the models on the original sample, using resampling [22] with 100 bootstrap samples to correct for optimistic bias. The optimistic bias of a model was estimated by calculating the mean and standard deviation of differences in model performance measures between the model developed on the original sample and developed on each bootstrap sample. The optimistic bias, for each performance measure, was added to the performances of the model developed on the original sample. The standard error of the optimistic bias was used to calculate the 95%-confidence interval of the performances. The proportional hazards assumption was not verified for CPH models that were developed on bootstrap samples. We considered a difference in performance between two models to be statistically significant if the bootstrap 95%-confidence interval of the difference in their performance did not contain zero. The 95%-confidence interval was calculated, taking the mean and standard deviation of the differences in performance between to models calculated for each bootstrap iteration.

Since the models we developed may perform differently for patients with a ICU LoS shorter or longer than four days and for patients with different main APACHE IV admission diagnosis categories, we estimated the performance for all patients and for these subgroups of patients separately.

Since the distribution of LoS is positively skewed and models for ICU LoS have differing capacity to predict this type of data, we not only estimated the performance of the models all patients, but also for the subgroups of patients with ICU LoS smaller than four days and greater than or equal to four days. This is roughly the 75% percentile of the ICU LoS distribution. Further, univariate analyses were performed to calculate R^2^ for the different patient characteristics to evaluate the contribution to the model. Finally, performance was calculated for the different main categories of the APACHE IV admission diagnosis, to investigate the performance for different diagnostic groups.

All statistical analyses were performed using R statistical software version 2.15.1 [23].

## Results

### Data

In 2011, data from 33,732 patients with medical or unplanned surgical ICU admissions satisfying APACHE IV inclusion criteria were recorded in the NICE database. Of these, 627 (3.2%) were subsequently discharged to an ICU in another hospital, eight (0.0%) had missing values for gender and 430 (2.4%) had missing values for GCS and were excluded. Hence, we included 32,667 patients in this study, of whom 28,280 (86.7%) were ICU survivors and 4,387 (13.3%) were ICU non-survivors.

**Figure 1:**
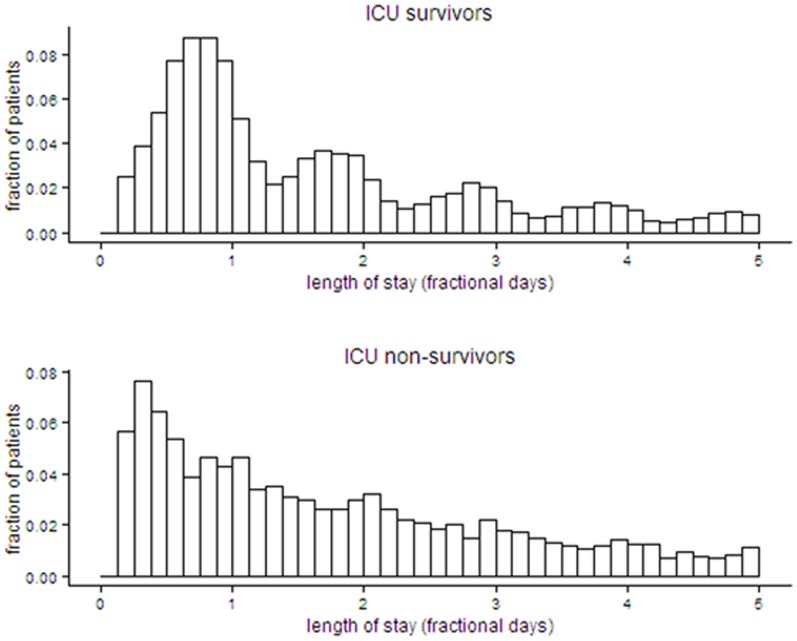
Distribution of ICU LoS for survivors and non-survivors.

Figure 1 shows the distribution of observed ICU LoS for the first five days of ICU admission for survivors and non-survivors. The distribution of ICU LoS was right skewed with a median of 1.7 (interquartile range (IQR) 0.8 to 4.0) days and a mean of 4.0 (standard deviation 7.6) days for survivors and a median and of 2.3 (IQR 0.9 to 6.0) days and a mean of 5.6 (standard deviation 9.8) days for non-survivors. For survivors the distribution of ICU LoS was multimodal.

Table 1 presents the demographics of the ICU survivors and non-survivors included in this study. Differences between survivors and non-survivors were tested using t-tests and χ^2^-tests and found to be statistically significant (gender p = 0.017, all other variables p<0.001). In Table S1, we summarize the patient characteristics, which remained in the models after stepwise backward selection of variables.

**Table 1:** Demographics of ICU admissions included in the analysis, for ICU survivors and ICU non-survivors separately (n = 32,667).

	ICU survivors	ICU non-survivors
Number of ICU admissions	28,280	4,387
ICU LoS in days, median (25%–75%)	1.7 (0.8–4.0)	2.3 (0.9–6.0)
ICU LoS in days, mean (sd)	4.0 (7.6)	5.6 (9.8)
Age in year, mean (sd)	60.7 (18.0)	68.6 (14.1)
Male (count %)	15,651 (55.1)	2,502 (57.0)
Admission type (count (%))		
Medical	20,903 (75.2)	3,537 (81.4)
Urgent surgery	7,377 (24.8)	850 (18.6)
APACHE IV APS, median (25%–75%)	44 (28–63)	95 (70–119)
Ventilation first 24 hours of ICU admission (count (%))	12,199 (42.2)	3,712 (84.5)
One or more chronic diagnoses (count (%))	19,463 (62.9)	4,137 (94.3)
One or more diagnoses at admission (<24 h) (count (%))	7,557 (26.9)	2,788 (63.8)
Confirmed infection (count (%))	5,922 (21.2)	1,302 (29.9)
Use of vasoactive drugs (count (%))	8,435 (29.5)	3,128 (71.5)
Lowest GCS first 24 hours, median (25%–75%)	15 (13–15)	6 (3–15)
Non-operative APACHE IV diagnosis category (count (%))		
Cardiovascular	5,932 (20.98)	1,740 (39.66)
Gastro-intestinal	1,630 (5.76)	244 (5.56)
Genito-uritary	705 (2.49)	52 (1.19)
Hematological	233 (0.81)	48 (1.09)
Metabolic	866 (3.06)	26 (0.59)
Musculoskeletal/skin	99 (0.35)	8 (0.18)
Neurological	4,214 (14.90)	408 (9.30
Respiratory	6,005 (21.23)	914 (20.83
Transplantation	7(0.02)	0 (0.00)
Trauma	1,209 (4.28)	97 (2.21)
Post-operative APACHE IV diagnosis category (count (%))		
Cardiovascular	2,248 (7.95)	378(8.62)
Gastro-intestinal	2,684 (9.49)	268 (6.11)
Genito-uritary	427 (1.51)	4 (0.09)
Hematological	4 (0.01)	0 (0.00)
Metabolic	12 (0.04)	0 (0.00)
Musculoskeletal/skin	324 (1.15)	9 (0.21)
Neurological	581 (2.05)	108 (2.46)
Respiratory	217 (0.77)	13 (0.30)
Transplantation	92 (0.33)	0 (0.00)
Trauma	795 (2.81)	70 (1.60)

LoS  =  Length of Stay, sd  =  standard deviation, GCS  =  Glasgow Coma scale.

For several patient characteristics, we found opposite associations with ICU LoS for survivors and non-survivors in all models. For instance, chronic dialysis resulted in a larger expected ICU LoS for non-survivors (OLS regression coefficient 1.91, (95% confidence interval −0.06 to 3.89) and a smaller expected ICU LoS for survivors (OLS regression coefficient −1.20, 95% confidence interval −1.97 to −0.44. The proportional hazards assumption was met for each of the CPH models that were developed on the entire dataset.

### Comparison of different regression methods

We present the estimates of predictive performance obtained from the bootstrap procedure in Table 2. We obtained values of R^2^ between 0.088 and 0.208, of RMSPE between 5.150 and 8.739 days, of MAPE between 3.004 and 3.927 days and of prediction bias between −2.993 and 0.030 days.

**Table 2:** Estimated performance of regression models, when using all patients for model construction and model validation, but no cyclical terms.

	No cyclical terms included			
	R^2^	Root mean squared prediction error (RMSPE)	Mean absolute prediction error (MAPE)	BIAS
OLS regression LoS	0.143	7.324	3.571	0.030
	(0.129 to 0.156)	(6.966 to 7.683)	(3.505 to 3.637)	(−0.051 to 0.112)
OLS regression LoS truncated at 30 days	0.208	5.150	3.099	0.015
	(0.2001 to 0.215)	(5.061 to 5.239)	(3.053 to 3.144)	(−0.044 to 0.074)
OLS regression log(LoS)	0.149	7.665	3.004	−1.850
	(0.132 to 0.166)	(7.302 to 8.029)	(2.928 to 3.080)	(−1.932 to −1.768)
GLM: Gaussian	0.154	7.279	3.431	−0.015
	(0.136 to 0.171)	(6.919 to7.640)	(3.366 to 3.496)	(−0.095 to 0.065)
GLM:Poisson	0.154	7.276	3.433	0.007
	(0.137 to 0.171)	(6.916 to 7.635)	(3.368 to 3.498)	(−0.073 to 0.086)
GLM: negative binomial	0.148	7.304	3.445	0.019
	(0.132 to 0.163)	(6.947 to 7.662)	(3.379 to 3.511)	(−0.061 to 0.100)
GLM: Gamma	0.147	7.306	3.446	0.020
	(0.132 to 0.163)	(6.948 to 7.663)	(3.380 to 3.512)	(−0.060 to 0.100)
Cox PH regression	0.088	8.739	3.927	−2.993
	(0.080 to 0.095)	(8.395 to 9.084)	(3.841 to 4.013)	(−3.083 to −2.903)
APACHE IV (original) LoS truncated at 30 days	0.163	5.546	4.103	1.640
	(0.156 to 0.169)	(5.470 to 5.621)	(4.063 to 4.144)	(1.579 to 1.700)
APACHE IV (recalibrated) LoS truncated at 30 days	0.169	5.291	3.375	0.366
	(0.162 to 0.175)	(5.204 to 5.379)	(3.331 to 3.420)	(0.305 to 0.427)

Confidence intervals were obtained with bootstrap sampling.

LoS  =  Length of Stay, OLS  =  Ordinary Least Square, GLM  =  General Linear Model.

The model predicting ICU LoS truncated at 30 days had the best performance. When considering R^2^, RMSPE and MAPE, the performance of the APACHE IV model is better than the models we developed. However, the prediction bias is more than one day for this model. Of the models which did not truncate LoS, the predictions made by the Poisson model and the Gaussian GLM had the largest values of R^2^ and smallest values of the RMSPE, and predictions made by the Poisson model had the smallest prediction bias, although the differences were not statistically significant. The values of R^2^ were significantly smaller and values for RMSPE, MAPE and prediction bias were significantly larger for CPH regression, compared to the values for the other models. Predictions with OLS regression had a large prediction bias and RMSPE, but small MAPE. The prediction bias for CPH regression and OLS regression of log-transformed LoS was negative, implying that these models systematically underestimate ICU LoS. We found a relatively large bias when using OLS regression on the log-transformed ICU LoS and comparing back-transformed predictions with observed ICU LoS. This bias is caused by the fact that we replaced negative predictions by zero and inflated when predicted values are back-transformed to the original scale.

We performed univariate GLM Poisson analyses to calculate R^2^ for each of the patient characteristics. The largest values for R^2^ were for mechanical ventilation in the first 24 hours of ICU stay (0.078), APS (0.067) and vasoactive medication (0.058).

We present the mean and standard deviation of the observed and predicted ICU LoS using the GLM Poisson model for a selection of common ICU diagnoses for ICU survivors and non-survivors in Table 3. Based on the R^2^ values, the models performed well for the categories operative metabolic, operative genito-uritary and non-operative trauma and poorly for post-operative neurological, post-operative musculoskeletal/skin and non-operative neurological.

**Table 3:** Observed and predicted ICU LoS (median (IQR)), for ICU survivors and ICU non-survivors.

APACHE IV diagnosis	ICU survivors		ICU non-survivors	
	Observed	Predicted	Observed	Predicted
Non-operative				
Cardiovascular	1.98 (0.95 to 4.31)	2.13 (1.09 to 4.04)	2.23 (0.93 to 4.72)	2.10 (1.48 to 3.04)
Gastro-intestinal	1.21 (0.74 to 2.77)	1.25 (0.92 to 2.08)	1.54 (0.72 to 4.38)	1.97 (1.25 to 2.74)
Genito-uritary	1.85 (0.88 to 3.53)	1.69 (1.19 to 2.71)	2.13 (1.04 to 5.46)	2.83 (1.85 to 3.81)
Metabolic	1.15 (0.75 to 2.04)	1.15 (0.90 to 1.54)	2.07 (0.85 to 5.89)	2.41 (1.63 to 3.43)
Musculoskeletal/skin	1.57 (0.71 to 3.27)	1.51 (0.99 to 2.48)	4.58 (1.51 to 8.23)	4.04 (2.66 to 5.47)
Neurological	0.91 (0.57 to 1.94)	0.97 (0.73 to 1.63)	1.28 (0.70 to 3.35)	1.42 (1.07 to 2.09)
Respiratory	2.59 (1.05 to 5.83)	2.54 (1.61 to 4.16)	3.73 (1.14 to 9.39)	3.69 (2.42 to 4.93)
Trauma	1.31 (0.70 to 2.91)	1.30 (1.05 to 2.22)	1.53 (0.44 to 8.02)	1.93 (1.42 to 2.79)
Post-operative				
Cardiovascular	2.31 (0.89 to 6.21)	2.69 (1.70 to 4.03)	2.53 (1.17 to 8.70)	3.49 (2.06 to 4.83)
Gastro-intestinal	1.62 (0.74 to 3.85)	1.75 (1.05 to 3.04)	2.23 (0.89 to 8.39)	3.08 (1.88 to 4.14)
Genito-uritary	0.79 (0.52 to 1.39)	0.78 (0.56 to 1.25)	4.07 (0.29 to 7.91)	1.38 (1.23 to 1.73)
Metabolic^1^	1.29 (0.84 to 2.26)	1.42 (0.94 to 1.54)		
Musculoskeletal/skin	0.90 (0.67 to 2.03)	1.05 (0.77 to 1.92)	4.16 (1.60 to 8.43)	3.08 (2.17 to 4.77)
Neurological	1.77 (0.89 to 5.57)	2.50 (1.35 to 3.70)	3.23 (1.20 to 5.56)	2.51 (1.99 to 3.81)
Respiratory	0.99 (0.72 to 3.51)	1.44 (0.97 to 1.99)	3.35 (3.15 to 5.78)	3.80 (3.02 to 4.67)
Trauma	1.26 (0.71 to 3.88)	1.57 (1.02 to 2.69)	2.45 (0.90 to 10.04)	2.49 (2.13 to 4.09)
Post- and non-operative				
Hematological^2^	1.19 (0.69 to 3.05)	1.42 (0.95 to 2.34)	4.83 (1.49 to 9.89)	4.37 (2.46 to 5.69)
Transplantation^3^	1.76 (1.10 to 2.85)	1.95 (1.34 to 2.43)		

Predictions were obtained using the Poisson model, constructed using all patients, but no cyclical terms.

1 *There were no non-survivors in the APACHE IV diagnose categories metabolic and transplantation.*

2 *Post- and non-operative hematological patients were combined because of the low number of post-operative hematological patients.*

3 *Post- and non-operative patients were combined because of the low number of non-operative patients with complications after transplantation.*

### Performance of separate models for survivors and non-survivors

Table 4 presents the estimated performance of separate models developed for survivors and non-survivors. In general, the performance of these models was better than the models for all patients. For survivors, the values for R^2^ were between 0.103 and 0.266, for RMSPE between 4.786 and 8.401 days, for MAPE between 2.701 and 3.754 and for the bias between −2.742 and 0.025 days. For non-survivors, the values of R^2^ were between 0.069 and 0.129, of RMSPE between 6.405 and 11.060, of MAPE between 4.325 and 5.165 and of the bias between −4.567 and 0.020 days.

**Table 4:** Estimated performance of regression models, when constructing models using ICU survivors and ICU non-survivors separately, but no cyclical terms.

	Survivors				non-survivors			
	R^2^	Root mean squared prediction error (RMSPE)	Mean absolute prediction error (MAPE)	BIAS	R^2^	Root mean squared prediction error (RMSPE)	Mean absolute prediction error (MAPE)	BIAS
OLS regression LoS	0.184	6.868	3.238	0.025	0.091	9.446	5.165	0.020
	(0.166 to 0.203)	(6.418 to 7.318)	(3.159 to 3.317)	(−0.059 to 0.109)	(0.074 to 0.109)	(8.687 to 10.205)	(4.941 to 5.389)	(−0.245 to 0.285)
OLS regression LoS truncated at 30 days	0.266	4.786	2.816	0.013	0.129	6.405	4.325	0.008
	(0.256 to 0.276)	(4.684 to 4.887)	(2.768 to 2.864)	(−0.042 to 0.069)	(0.109 to 0.149)	(6.178 to 6.632)	(4.195 to 4.455)	(−0.169 to 0.184)
OLS regression log (LoS)	0.196	7.135	2.701	−1.584	0.094	9.969	4.369	−2.892
	(0.174 to 0.217)	(6.675 to 7.596)	(2.619 to 2.783)	(−1.669 to −1.499)	(0.074 to 0.113)	(9.136 to 10.801)	(4.112 to 4.627)	(−3.161 to 2.623)
GLM: Gaussian	0.202	6.797	3.063	−0.033	0.094	9.470	5.027	−0.130
	(0.179 to 0.224)	(6.348 to 7.246)	(2.985 to 3.141)	(−0.115 to 0.050)	(0.059 to 0.128)	(8.742 to 10.199)	(4.800 to 5.255)	(−0.389 to 0.129)
GLM: Poisson	0.202	6.793	3.061	−0.007	0.098	9.412	5.062	−0.006
	(0.180 to 0.224)	(6.341 to 7.245)	(2.983 to 3.139)	(−0.090 to 0.075)	(0.072 to 0.124)	(8.666 to 10.158)	(4.837 to 5.287)	(−0.261 to 0.249)
GLM: negative binomial	0.196	6.821	3.068	−0.010	0.097	9.418	5.063	0.003
	(0.175 to 0.216)	(6.370 to 7.272)	(2.989 to 3.146)	(−0.092 to 0.073)	(0.077 to 0.117)	(8.665 to 10.172)	(4.837 to 5.288)	(−0.253 to 0.260)
GLM: Gamma	0.196	6.821	3.068	−0.010	0.097	9.422	5.066	0.004
	(0.175 to 0.216)	(6.371 to 7.272)	(2.990 to 3.146)	(−0.092 to 0.073)	(0.077 to 0.117)	(8.668 to 10.176)	(4.840 to 5.292)	(−0.252 to 0.261)
Cox PH regression	0.103	8.401	3.754	−2.742	0.069	11.060	5.137	−4.567
	(0.093 to 0.113)	(7.975 to 8.827)	(3.662 to 3.847)	(−2.838 to −2.647)	(0.059 to 0.080)	(10.227 to 11.893)	(4.848 to 5.426)	(−4.861 to −4.272)

Confidence intervals were obtained using bootstrap sampling.

LoS  =  Length of Stay, OLS  =  Ordinary Least Square, GLM  =  General Linear Model.

Generally speaking, the models for ICU survivors performed better than the models for ICU non-survivors. Furthermore, as before, the best results for R^2^ and the RMSPE were obtained with GLMs, in particular the Gaussian GLM and the Poisson model, but the Gaussian model resulted in a relatively large prediction bias. The predictions obtained from the CPH model and OLS model of log transformed ICU LoS exhibited a large prediction bias.

### Cyclical terms

Table 5 shows the results we obtained when we included the cyclical terms for discharge time in the models. In general, the values of R^2^ and prediction bias were higher and the values of the RMSPE, the MAPE and the bias were smaller when we included the cyclical terms.

**Table 5:** Estimated performance of regression models, when constructing models using all patients and cyclical terms.

	Cyclical terms included			
	R^2^	Root mean squared prediction error (RMSPE)	Mean absolute prediction error (MAPE)	BIAS
OLS regression (LoS)	0.144	7.317	3.566	0.034
	(0.131 to 0.157)	(6.958 to 7.675)	(3.500 to 3.631)	(−0.048 to 0.115)
OLS regression LoS truncated at 30 days	0.210	5.141	3.093	0.018
	(0.203 to 0.218)	(5.053 to 5.229)	(3.049 to 3.138)	(−0.041 to 0.078)
OLS regression log(LoS)	0.150	7.647	2.991	−1.827
	(0.135 to 0.164)	(7.283 to 8.012)	(2.916 to 3.067)	(−1.909 to −1.745)
GLM: Gaussian	0.156	7.270	3.424	−0.018
	(0.138 to 0.174)	(6.909 to 7.632)	(3.360 to 3.489)	(−0.098 to 0.061)
GLM: Poisson	0.157	7.264	3.424	0.007
	(0.141 to 0.173)	(6.905 to 7.623)	(3.359 to 3.489)	(−0.073 to 0.086)
GLM: negative binomial	0.151	7.288	3.435	0.017
	(0.137 to 0.165)	(6.925 to 7.651)	(3.369 to 3.501)	(−0.063 to 0.098)
GLM: Gamma	0.151	7.286	3.436	0.017
	(0.137 to 0.165)	(6.908 to 7.664)	(3.369 to 3.502)	(−0.063 to 0.097)
Cox PH regression	0.086	8.743	3.939	−2.985
	(0.079 to 0.094)	(8.399 to 9.087)	(3.853 to 4.024)	(−3.075 to −2.895)

Confidence intervals were obtained with bootstrap sampling.

LoS  =  Length of Stay, OLS  =  Ordinary Least Square, GLM  =  General Linear Model.

### Performance for patients with short and long ICU LoS

Table 6 presents the predictive performance of each of the regression models, separately for patients with an ICU LoS less or more than four days. The models are the same as those presented in Table 1 in that they were developed using data from all patients and without cyclical terms for discharge time.

**Table 6:** Performance measures using all patients for model prediction, but not including cyclical terms as covariate separated, for patients with length of stay smaller than the 75% percentile and larger or equal than the 75% percentile for validation.

	ICU LoS smaller than 75% percentile				ICU LoS larger or equal to the 75% percentile			
	R^2^	Root mean squared prediction error (RMSPE)	Mean absolute prediction error (MAPE)	BIAS	R^2^	Root mean squared prediction error (RMSPE)	Mean absolute prediction error (MAPE)	BIAS
OLS regression LoS	0.124	3.208	2.391	2.012	0.038	13.578	7.129	−5.945
	(0.114 to 0.134)	(3.164 to 3.252)	(2.364 to 2.418)	(1.980 to 2.045)	(0.024 to 0.052)	(12.800 to 14.355)	(6.873 to 7.385)	(−6.245 to −5.645)
OLS regression LoS truncated at 30 days	0.130	2.826	2.124	1.709	0.063	9.078	6.037	−5.273
	(0.121 to 0.139)	(2.798 to 2.854)	(2.101 to 2.147)	(1.740 to 1.798)	(0.053 to 0.073)	(8.874 to 9.282)	(5.868 to 6.206)	(−5.482 to −5.063)
OLS regression log(LoS)	0.132	1.429	1.010	0.497	0.033	15.157	9.015	−8.924
	(0.120 to 0.144)	(1.393 to 1.465)	(0.995 to 1.025)	(0.476 to 0.517)	(0.020 to 0.046)	(14.417 to 15.896)	(8.717 to 9.313)	(−9.230 to −8.618)
GLM: Gaussian	0.112	3.122	2.137	1.903	0.040	13.540	7.333	−5.798
	(0.102 to 0.121)	(3.068 to 3.176)	(2.107 to 2.166)	(1.870 to 1.936)	(0.024 to 0.057)	(12.763 to 14.317)	(7.086 to 7.581)	(−6.090 to −5.506)
GLM: Poisson	0.109	3.119	2.147	1.938	0.044	13.357	7.273	−5.769
	(0.100 to 0.119)	(3.065 to 3.172)	(2.118 to 2.176)	(1.905 to 1.971)	(0.029 to 0.058)	(12.705 to 14.008)	(7.031 to 7.516)	(−6.051 to −5.487)
GLM: negative binomial	0.106	3.175	2.156	1.952	0.041	13.378	7.294	−5.759
	(0.097 to 0.115)	(3.121 to 3.229)	(2.126 to 2.186)	(1.919 to 1.985)	(0.028 to 0.054)	(12.727 to 14.030)	(7.050 to 7.538)	(−6.045 to −5.474)
GLM: Gamma	0.106	3.177	2.157	1.952	0.038	13.559	7.330	−5.803
	(0.097 to 0.116)	(3.123 to 3.231)	(2.128 to 2.187)	(1.919 to 1.985)	(0.024 to 0.052)	(12.786 to 14.331)	(7.079 to 7.580)	(−6.099 to −5.507)
Cox (PH) regression	0.135	1.593	1.269	−0.023	0.020	17.261	11.903	−11.903
	(0.127 to 0.143)	(1.569 to 1.617)	(1.251 to 1.286)	(−0.051 to 0.006)	(0.011 to 0.028)	(16.565 to 17.957)	(11.589 to 12.218)	(−12.218 to −11.589)
APACHE IV (original) LoS truncated at 30 days	0.094	4.079	3.636	3.601	0.053	8.551	5.506	−4.245
	(0.087 to 0.102)	(4.051 to 4.107)	(3.608 to 3.664)	(3.572 to 3.630)	(0.044 to 0.062)	(8.353 to 8.749)	(5.351 to 5.661)	(−4.452 to −4.037)
APACHE IV (recalibrated) LoS truncated at 30 days	0.096	3.176	2.499	2.202	0.053	9.042	6.003	−5.142
	(0.088 to 0.104)	(3.145 to 3.207)	(2.473 to 2.526)	(2.168 to 2.235)	(0.044 to 0.062)	(8.837 to 9.246)	(5.833 to 6.174)	(−5.348 to −4.937)

LoS  =  Length of Stay, OLS  =  Ordinary Least Square, GLM  =  General Linear Model.

For patients ICU LoS of less than four days, we obtained the best predictions using CPH regression; OLS regression on LoS truncated at 30 days and OLS regression of log-transformed ICU LoS. These models had better results for R^2^, RMSPE, MAPE and prediction bias, than the other models. For patients with an ICU LoS of longer than four days, we obtained the best values for R^2^ the Poisson model. For these patients, we obtained the worst values of R^2^, RMSPE, MAPE and prediction bias from CPH regression and OLS regression on log-transformed LoS. When separate models were developed for survivors and non-survivors separately, see respectively Table S2 and S3 and when cyclical terms were included for discharge time, this gave similar findings, see Table S4.

## Discussion

In this study, we compared regression methods for predicting LoS for unplanned ICU admissions on a large registry dataset. As expected, the distribution of ICU LoS in our dataset was extremely skewed to the right. In addition, the ICU mortality among the patients in our dataset was substantial (12%), and ICU LoS was generally longer for survivors than non-survivors. Furthermore, there were considerable differences in observed ICU LoS for different APACHE IV diagnoses.

The predictive performance of all of our models was disappointing, with an R^2^ at most around 20% and a RMSPE of more than seven days. Even in absolute terms, our predictions were, on average, three days different from the observed ICU LoS. Given that more than half of the patients had an ICU LoS of less than two days, it is fair to say that these predictions are not particularly useful. The differences in predictive performance between the models were generally small. Overall, the Poisson model and Gaussian GLM performed somewhat better than the other models, while CPH regression and OLS regression of log-transformed ICU LoS were superior for patients with an ICU LoS of less than four days. The models generally performed better for ICU survivors than for non-survivors. Because patients are often discharged at set times during the day, we hypothesized that the inclusion of cyclical terms for discharge times would improve the performance of the models. However, the performance only improved marginally after we included these terms.

ICU discharge decisions often do not only depend on a patient's recovery, but on organizational circumstances such as availability of beds on the general ward and the need to free up ICU beds for other patients. These organizational circumstances depend on structural factors related to the ICU and the hospital. We have deliberately chosen not to include ICU and hospital level covariates in our models, because we wished to investigate the feasibility of predicting ICU LoS for future use in tools to compare ICUs [24].

Previously researchers have used regression models to predict ICU LoS, but they have generally not critically appraised and compared the performance of different models [6], [9], [11]–[13], [25]. The APACHE IV model for predicting ICU LoS uses OLS regression on ICU LoS truncated at 30 days. We have shown (Table 4) that this model leads to biased results for patients admitted to the ICU for less than four days, but performs better for patients with ICU LoS longer than four days, perhaps due to truncation. Other researchers have used CPH regression to predict ICU LoS following cardiac surgery and have found that their models were able to discriminate between shorter and longer treatment durations, but were unsuitable for predictions in individual patients [25]. Researchers examining hospital LoS following CABG surgery found that the model assumptions for linear regression were not satisfied for LoS or log transformed LoS and conclude that the use of GLMs with a logarithmic link function should be considered for this type of data [15]. Another study compared twelve methods to estimate ICU LoS using a cohort of patients in Australian and New Zealand [27]. These researchers compared OLS regression on log-transformed ICU LoS; GLMs with a log-link function (distributions Poisson, gamma, negative binomial and inverse-Gaussian); linear mixed models; skew-normal; skew-t models; extended estimating equations and a finite mixture model. They obtained values for R^2^ between 0.17 and 0.22 and found that linear mixed models and OLS regression on log-transformed LoS performed best.

Because ICU LoS is right skewed, OLS regression is theoretically not a good choice. Researchers have suggested truncating observed ICU LoS, to improve the performance of OLS regression [6], [9], [11]–[13]. In this study, we used OLS regression on ICU LoS truncated at 30 days (Table 2). However, when comparing ICU LoS among hospitals, truncation of ICU LoS can be unfair because there may be substantial differences in the values that were truncated and the largest improvements in efficiency can probably be achieved in patients with the longest ICU LoS.

The predictive performance of separate models for survivors and non-survivors was higher than for combined models. This may be caused by differences in the signs of regression coefficients between survivors and non-survivors. Factors that aggravate illness severity tend to increase the length of stay for survivors and shorten it for non-survivors. Nevertheless, a fundamental drawback is that some hospitals may achieve shorter average ICU LoS because their mortality rates are higher, which again would make the comparison unfair. Furthermore, this could explain partly the poor performance of the models.

Compared to previously published studies, our work stands out because we applied resampling methods to compare eight types of models constructed using advanced modelling methods, such as regression splines and cyclical terms, and we based our study on a large multi-center dataset. Furthermore, we used cyclic terms of time of discharge as a way to model center effects in models with patients variables only. This approach has not been used to predict ICU LoS previously. Yet our work also has a number of limitations. First, we did not evaluate modern prediction methods from the field of statistical learning, such as ensemble [26] and kernel methods [27]. As a result, we have not explored all methods for predicting ICU LoS, which could be done in future research. Second, we had no information on how the logistic policies vary between the ICUs included in our study. For example, some ICUs usually discharged patients in the morning, while others do this in the afternoon. Thirdly, for this study we did not include any interaction terms in our models. Developing a model to predict ICU LoS may require more accurate analyses on the role of interaction terms in the model. Fourth, we did not include predictions of LoS for elective surgery patients in this study. Patients undergoing unplanned ICU-admission differ considerably from those undergoing elective surgery and often have a protocolized ICU LoS. Hence, we choose not to develop a single model for patients with planned and unplanned ICU admissions. Fitfth, interestingly, many patients in our cohort of unplanned ICU-admissions had a very short LoS on the ICU. Of our patients 25% had an ICU LoS shorter than 0.8 days. This group of patients included both medical and unplanned surgical patients and many different reasons for ICU admission were present, such as monitoring during endoscopic procedures and monitoring after overdose of sedatives. Some patients only required short ICU treatment for respiratory failure, e.g. pulmonary edema well responding to administration of diuretics. Also, LoS could be very short in patients who died within the first hours after ICU admission. We do not expect that the shorter LoS in our population influences our conclusions regarding the preferred regression model for length of stay as the shape of the LoS distribution in other ICU populations is comparable. Sixth, the performance of the CPH models may have been underestimated, because we did not verify the proportional hazards assumption for models developed within the bootstrap procedure. However, we believe that violations of this assumption were unlikely, as it was satisfied for all models that were developed on the entire dataset.

Our findings have implications for the use of patient level predictions of ICU LoS. We believe that currently available models for ICU LoS, are unsuitable for use in quality indicators and that further research is needed to develop models of ICU LoS. A relatively small group of patients determines the variation in ICU LoS, but it is extremely difficult to identify these patients. We are not sure whether observed differences in ICU LoS are due to variations in the quality of care. Therefore we advise against using currently available models for ICU LoS in unplanned ICU admissions as input for policy development or evaluation [6], [9].

## Conclusions

It is difficult to predict ICU LoS for patients with unplanned admissions using patient characteristics at ICU admission time only, even with sophisticated statistical modelling methods. Although the differences were small, GLMs with a logarithmic link function predicted ICU LoS slightly better than other models for untransformed ICU LoS. For patients with ICU LoS shorter than four days, CPH regression and OLS regression of log-transformed LoS were superior. All models performed only marginally better when we included cyclical terms for discharge time. Models developed using survivors and non-survivors separately performed better than models developed on data for all patients. We conclude that currently available models for ICU LoS are not suitable for predicting individual patient data and should not be used as an indicator for ICU quality or efficiency or as tools to develop policies around unplanned ICU admissions.

## Supporting Information

Table S1 **Variables included in the regression models after performing stepwise backward selection of variables.**
(XLS)

Table S2 **Performance measures using ICU survivors for model prediction, but not including cyclical terms as covariate separated, for patients with length of stay smaller than the 75% percentile and larger or equal than the 75% percentile for validation.**
(DOC)

Table S3 **Performance measures using ICU non-survivors for model prediction, but not including cyclical terms as covariate separated, for patients with length of stay smaller than the 75% percentile and larger or equal than the 75% percentile for validation.**
(DOC)

Table S4 **Performance measures using all patients for model prediction and cyclical terms as covariate separated, for patients with length of stay smaller than the 75% percentile and larger or equal than the 75% percentile for validation.**
(DOC)

Dataset S1 **Description of patient characteristics used to perform the analyses.**
(XLS)

Text S1 **Statistical regression methods.**
(DOC)

Text S2 **Cyclical terms included as covariate in the models.**
(DOC)

Text S3 **Performance assessment of the models developed.**
(DOC)
