# Supplementary material for: Comparison of Regression Methods for Modeling Intensive Care Length of Stay
Source: PLoS One. 2014 Oct 31;9(10):e109684. doi: 10.1371/journal.pone.0109684 (PMC4215850; doi:10.1371/journal.pone.0109684)
Supplement: Table S2 — Performance measures using ICU survivors for model prediction, but not including cyclical terms as covariate separated, for patients with length of stay smaller than the 75% percentile and larger or equal than the 75% percentile for validation. (DOC) [file pone.0109684.s002.doc]

**Table S2. Performance measures using ICU survivors for model prediction, but not including cyclical terms as covariate separated, for patients with length of stay smaller than the 75% percentile and larger or equal than the 75% percentile for validation.**

|  | ICU LoS smaller than 75% percentile | | | | ICU LoS larger or equal to the 75% percentile | | | |
| --- | --- | --- | --- | --- | --- | --- | --- | --- |
|  | R² | Root mean squared prediction error (RMSPE) | Mean absolute prediction error (MAPE) | BIAS | R² | Root mean squared prediction error (RMSPE) | Mean absolute prediction error (MAPE) | BIAS |
| OLS regression LoS | 0.153 | 2.934 | 2.112 | 1.667 | 0.052 | 12.714 | 6.581 | -4.851 |
|  | (0.142 to 0.165) | (2.892 to 2.976) | (2.087 to 2.138) | (1.635 to 1.700) | (0.037 to 0.067) | (11.752 to 13.676) | (6.289 to 6.872) | (-5.177 to -4.526) |
| OLS regression LoS truncated at 30 days | 0.160 | 2.601 | 1.897 | 1.483 | 0.086 | 8.415 | 5.544 | -4.349 |
|  | (0.150 to 0.171) | (2.571 to 2.630) | (1.874 to 1.920) | (1.453 to 1.513) | (0.075 to 0.097) | (8.190 to 8.639) | (5.375 to 5.714) | (-4.553 to -4.145) |
| OLS regression log(LoS) | 0.155 | 1.409 | 0.943 | 0.450 | 0.052 | 14.006 | 7.923 | -7.622 |
|  | (0.143 to 0.167) | (1.374 to 1.444) | (0.929 to 0.957) | (0.430 to 0.471) | (0.038 to 0.067) | (13.077 to 14.935) | (7.607 to 8.238) | (-7.951 to -7.293) |
| GLM: Gaussian | 0.139 | 2.774 | 1.809 | 1.538 | 0.060 | 12.669 | 6.784 | -4.695 |
|  | (0.128 to 0.150) | (2.716 to 2.833) | (1.784 to 1.835) | (1.508 to 1.569) | (0.041 to 0.079) | (11.714 to 13.624) | (6.497 to 7.071) | (-5.018 to -4.372) |
| GLM: Poisson | 0.141 | 2.747 | 1.810 | 1.574 | 0.059 | 12.679 | 6.775 | -4.701 |
|  | (0.130 to 0.152) | (2.691 to 2.803) | (1.785 to 1.835) | (1.544 to 1.603) | (0.042 to 0.076) | (11.719 to 13.639) | (6.485 to 7.064) | (-5.023 to -4.380) |
| GLM: negative binomial | 0.144 | 2.758 | 1.804 | 1.581 | 0.054 | 12.732 | 6.819 | -4.731 |
|  | (0.132 to 0.155) | (2.701 to 2.814) | (1.779 to 1.829) | (1.551 to 1.610) | (0.039 to 0.069) | (11.774 to 13.690) | (6.528 to 7.109) | (-5.052 to -4.409) |
| GLM: Gamma | 0.144 | 2.758 | 1.804 | 1.581 | 0.054 | 12.732 | 6.819 | -4.731 |
|  | (0.132 to 0.155) | (2.701 to 2.815) | (1.779 to 1.830) | (1.551 to 1.610) | (0.039 to 0.069) | (11.775 to 13.689) | (6.529 to 7.108) | (-5.052 to -4.409) |
| Cox (PH) regression | 0.158 | 1.556 | 1.248 | 0.105 | 0.025 | 16.520 | 11.196 | -11.196 |
|  | (0.150 to 0.166) | (1.534 to 1.578) | (1.230 to 1.265) | (0.080 to 0.131) | (0.015 to 0.036) | (15.663 to 17.377) | (10.844 to 11.548) | (-11.548 to -10.844) |

LoS = Length of Stay, OLS = Ordinary Least Square, GLM = General Linear Model
