# Supplementary material for: Comparison of Regression Methods for Modeling Intensive Care Length of Stay
Source: PLoS One. 2014 Oct 31;9(10):e109684. doi: 10.1371/journal.pone.0109684 (PMC4215850; doi:10.1371/journal.pone.0109684)
Supplement: Table S3 — Performance measures using ICU non-survivors for model prediction, but not including cyclical terms as covariate separated, for patients with length of stay smaller than the 75% percentile and larger or equal than the 75% percentile for validation. (DOC) [file pone.0109684.s003.doc]

**Table S3.** **Performance measures using ICU non-survivors for model prediction, but not including cyclical terms as covariate separated, for patients with length of stay smaller than the 75% percentile and larger or equal than the 75% percentile for validation.**

|  | ICU LoS smaller than 75% percentile | | | | ICU LoS larger or equal to the 75% percentile | | | |
| --- | --- | --- | --- | --- | --- | --- | --- | --- |
|  | R² | Root mean squared prediction error (RMSPE) | Mean absolute prediction error (MAPE) | BIAS | R² | Root mean squared prediction error (RMSPE) | Mean absolute prediction error (MAPE) | BIAS |
| OLS regression LoS | 0.060 | 4.408 | 3.541 | 3.155 | 0.031 | 17.258 | 10.021 | -9.353 |
|  | (0.044 to 0.076) | (4.312 to 4.504) | (3.452 to 3.629) | (3.048 to 3.263) | (0.003 to 0.058) | (15.598 to 18.918) | (9.137 to 10.906) | (-10.298 to -8.408) |
| OLS regression LoS truncated at 30 days | 0.065 | 3.714 | 3.013 | 2.671 | 0.042 | 11.063 | 8.247 | -7.956 |
|  | (0.047 to 0.083) | (3.637 to 3.791) | (2.940 to 3.086) | (2.575 to 2.768) | (0.011 to 0.072) | (10.533 to 11.593) | (7.734 to 8.760) | (-8.512 to -7.400) |
| OLS regression log(LoS) | 0.081 | 1.847 | 1.421 | 0.546 | 0.022 | 19.654 | 13.184 | -13.172 |
|  | (0.060 to 0.103) | (1.788 to 1.906) | (1.377 to 1.465) | (0.464 to 0.629) | (0.003 to 0.040) | (17.974 to 21.334) | (12.208 to 14.159) | (-14.149 to -12.195) |
| GLM: Gaussian | 0.054 | 4.393 | 3.240 | 2.906 | 0.024 | 17.322 | 10.370 | -9.206 |
|  | (0.038 to 0.070) | (4.214 to 4.572) | (3.132 to 3.349) | (2.782 to 3.030) | (-0.027 to 0.074) | (15.742 to 18.902) | (9.529 to 11.211) | (-10.139 to -8.274) |
| GLM: Poisson | 0.056 | 4.352 | 3.340 | 3.074 | 0.028 | 17.226 | 10.211 | -9.214 |
|  | (0.040 to 0.072) | (4.223 to 4.480) | (3.246 to 3.434) | (2.963 to 3.184) | (-0.008 to 0.065) | (15.602 to 18.849) | (9.354 to 11.068) | (-10.141 to -8.287) |
| GLM: negative binomial | 0.060 | 4.344 | 3.343 | 3.090 | 0.028 | 17.247 | 10.206 | -9.225 |
|  | (0.044 to 0.076) | (4.226 to 4.462) | (3.251 to 3.434) | (2.981 to 3.198) | (0.002 to 0.053) | (15.603 to 18.891) | (9.336 to 11.076) | (-10.154 to -8.295) |
| GLM: Gamma | 0.060 | 4.352 | 3.349 | 3.095 | 0.029 | 17.248 | 10.201 | -9.237 |
|  | (0.044 to 0.076) | (4.233 to 4.470) | (3.257 to 3.440) | (2.987 to 3.204) | (0.004 to 0.054) | (15.604 to 18.893) | (9.331 to 11.071) | (-10.168 to -8.306) |
| Cox (PH) regression | 0.078 | 1.937 | 1.488 | -0.727 | 0.022 | 21.837 | 16.047 | -16.045 |
|  | (0.063 to 0.093) | (1.809 to 2.065) | (1.404 to 1.571) | (-0.827 to -0.628) | (0.003 to 0.041) | (20.164 to 23.510) | (15.022 to 17.073) | (-17.073 to -15.016) |

LoS = Length of Stay, OLS = Ordinary Least Square, GLM = General Linear Model
