# Supplementary material for: Comparison of Regression Methods for Modeling Intensive Care Length of Stay
Source: PLoS One. 2014 Oct 31;9(10):e109684. doi: 10.1371/journal.pone.0109684 (PMC4215850; doi:10.1371/journal.pone.0109684)
Supplement: Table S4 — Performance measures using all patients for model prediction and cyclical terms as covariate separated, for patients with length of stay smaller than the 75% percentile and larger or equal than the 75% percentile for validation. (DOC) [file pone.0109684.s004.doc]

**Table S4. Performance measures using all patients for model prediction and cyclical terms as covariate separated, for patients with** length of stay smaller than the 75% percentile and larger or equal than the 75% percentile for validation.

|  | ICU LoS smaller than 75% percentile | | | | ICU LoS larger or equal to the 75% percentile | | | |
| --- | --- | --- | --- | --- | --- | --- | --- | --- |
|  | R² | Root mean squared prediction error (RMSPE) | Mean absolute prediction error (MAPE) | BIAS | R² | Root mean squared prediction error (RMSPE) | Mean absolute prediction error (MAPE) | BIAS |
| OLS regression LoS | 0.137 | 3.194 | 2.378 | 2.007 | 0.038 | 13.567 | 7.117 | -5.915 |
|  | (0.127 to 0.148) | (3.150 to 3.239) | (2.351 to 2.405) | (1.975 to 2.040) | (0.024 to 0.052) | (12.789 to 14.345) | (6.862 to 7.373) | (-6.215 to -5.615) |
| OLS regression LoS truncated at 30 days | 0.139 | 2.814 | 2.119 | 1.765 | 0.062 | 9.068 | 6.031 | -5.247 |
|  | (0.130 to 0.148) | (2.786 to 2.842) | (2.096 to 2.142) | (1.736 to 1.795) | (0.052 to 0.072) | (8.864 to 9.271) | (5.862 to 6.200) | (-5.456 to -5.039) |
| OLS regression log(LoS) | 0.154 | 1.444 | 1.011 | 0.511 | 0.029 | 15.110 | 8.952 | -8.822 |
|  | (0.140 to 0.168) | (1.399 to 1.489) | (0.995 to 1.026) | (0.489 to 0.533) | (0.017 to 0.040) | (14.366 to 15.855) | (8.656 to 9.249) | (-9.127 to -8.517) |
| GLM: Gaussian | 0.121 | 3.096 | 2.122 | 1.890 | 0.039 | 13.539 | 7.342 | -5.754 |
|  | (0.111 to 0.131) | (3.040 to 3.152) | (2.092 to 2.152) | (1.856 to 1.924) | (0.022 to 0.055) | (12.759 to 14.318) | (7.094 to 7.590) | (-6.045 to -5.463) |
| GLM: Poisson | 0.122 | 3.091 | 2.129 | 1.922 | 0.040 | 13.535 | 7.314 | -5.766 |
|  | (0.112 to 0.132) | (3.034 to 3.149) | (2.099 to 2.159) | (1.889 to 1.955) | (0.024 to 0.055) | (12.757 to 14.314) | (7.063 to 7.564) | (-6.059 to -5.473) |
| GLM: negative binomial | 0.121 | 3.172 | 2.144 | 1.942 | 0.036 | 13.555 | 7.343 | -5.721 |
|  | (0.111 to 0.131) | (3.112 to 3.233) | (2.113 to 2.175) | (1.908 to 1.975) | (0.023 to 0.050) | (12.778 to 14.332) | (7.093 to 7.594) | (-6.017 to -5.424) |
| GLM: Gamma | 0.122 | 3.173 | 2.145 | 1.942 | 0.036 | 13.557 | 7.346 | -5.720 |
|  | (0.111 to 0.132) | (3.113 to 3.233) | (2.114 to 2.176) | (1.909 to 1.976) | (0.023 to 0.050) | (12.780 to 14.334) | (7.095 to 7.597) | (-6.016 to -5.424) |
| Cox (PH) regression | 0.150 | 1.673 | 1.316 | 0.007 | 0.019 | 17.294 | 11.937 | -11.937 |
|  | (0.142 to 0.158) | (1.648 to 1.697) | (1.297 to 1.335) | (-0.024 to 0.037) | (0.011 to 0.027) | (16.592 to 17.996) | (11.613 to 12.261) | (-12.260 to -11.613) |

LoS = Length of Stay, OLS = Ordinary Least Square, GLM = General Linear Model
