# Supplementary material for: Comparison of Regression Methods for Modeling Intensive Care Length of Stay
Source: PLoS One. 2014 Oct 31;9(10):e109684. doi: 10.1371/journal.pone.0109684 (PMC4215850; doi:10.1371/journal.pone.0109684)
Supplement: Text S1 — Statistical regression methods. (DOC) [file pone.0109684.s006.doc]

**Text S1: Statistical regression methods**

The GLM with a Gaussian distribution and a logarithmic link function differs from OLS regression on log-transformed ICU LoS because the former regresses on the log-transformed expected LoS while the latter regresses on the expected log-transformed LoS [1]. Skewness of the distribution of the outcome variable and heteroscedasticity can lead to bias when using OLS regression with log transformed dependent variable and can result in a loss of precision when using GLM [1]. It has been suggested that truncating ICU LoS at 30 days will improve performance [2].

The advantage of Poisson, negative binomial and Gamma regression is that their response distributions are positively skewed, and are therefore expected to be well suited for modeling LoS. Several studies showed that Gamma regression can be used to predict LoS [3-5]. A limitation of the Poisson distribution is that its expectation equals its variance, which can lead to overdispersion, defined as there being more variance in the observed data than in the predictions generated by the model. Therefore we also examined the negative binomial distribution, which is a generalization of the Poisson distribution with a separate parameter for estimating the variance.

The final method, CPH regression, models the probability of ICU discharge as a function of time instead of modeling the distribution of ICU LoS itself. The main advantages of CPH regression is that this function is modeled non-parametrically and hence imposes no assumptions on the shape of the LoS distribution.

Reference List

[1] Manning WG, Mullahy J. (2001 Jul) Estimating log models: to transform or not to transform? J Health Econ 20 (4): 461-94.

[2] Zimmerman JE, Kramer AA, McNair DS, Malila FM. (2006 May) Acute Physiology and Chronic Health Evaluation (APACHE) IV: hospital mortality assessment for today's critically ill patients. Crit Care Med 34 (5): 1297-310.

[3] Faddy M, Graves N, Pettitt A. (2009 Mar) Modeling length of stay in hospital and other right skewed data: comparison of phase-type, gamma and log-normal distributions. Value Health.

[4] Lee AH, Wang K, Yau KK, McLachlan GJ, Ng SK. (2007 Aug) Maternity length of stay modelling by gamma mixture regression with random effects. Biom J.

[5] Straney L, Clements A, Alexander J, Slater A. (2010) Quantifying variation of paediatric length of stay among intensive care units in Australia and New Zealand. Qual Saf Health Care.
