# Supplementary material for: Comparison of Regression Methods for Modeling Intensive Care Length of Stay
Source: PLoS One. 2014 Oct 31;9(10):e109684. doi: 10.1371/journal.pone.0109684 (PMC4215850; doi:10.1371/journal.pone.0109684)
Supplement: Text S2 — Cyclical terms included as covariate in the models. (DOC) [file pone.0109684.s007.doc]

**Text S2. cyclical terms included as covariate in the models**

Cyclical terms were included as cosine function:

$f(t)=\alpha\cdot\cos\left[\frac{2\cdot\pi\cdot t}{24}-\theta\right],$ (1)
where *t* was the discharge time expressed in hours, *θ* was the horizontal shift (time delay) in the cosine function and *α* was its amplitude. As *θ* is unknown, the cosine function has to be transformed as a combination of a cosine and sine function, such that regression could be performed:

$f(t)=\beta_{1}\cdot\sin\left[\frac{2\cdot\pi\cdot t}{24}\right]+\beta_{2}\cdot\cos\left[\frac{2\cdot\pi\cdot t}{24}\right]$, (2)

with *β_1_=α∙*cos(*θ*) and *β_2=_ α∙*sin(*θ*) These sine and cosine terms were for both discharge time included as covariates in the models [1]. Parameters *β_1_* and *β_2_* were estimated by the different regression methods used.

Reference List

[1] Stolwijk AM, Straatman H, Zielhuis GA. (1999 Apr) Studying seasonality by using sine and cosine functions in regression analysis. J Epidemiol Community Health 53 (4): 235-8.
