# Supplementary material for: Comparison of Regression Methods for Modeling Intensive Care Length of Stay
Source: PLoS One. 2014 Oct 31;9(10):e109684. doi: 10.1371/journal.pone.0109684 (PMC4215850; doi:10.1371/journal.pone.0109684)
Supplement: Text S3 — Performance assessment of the models developed. (DOC) [file pone.0109684.s008.doc]

**Text S3. Performance assessment of the models developed**

Performance measures were defined as follows:

(4)

, (5)

(6)

(7)

Here, *n* is the number of patients in the dataset, *y_i_* the observed ICU LoS for patient *i*, the predicted ICU LoS for patient *i*, and Cov*(Y,Ŷ)*, *σ(Y)* and *σ(Ŷ)* are respectively the covariance and standard deviations of the vector of observations *Y* and the vector of predictions *Ŷ*.
